# Tracing the Origins of Autism: A Spectrum of New Studies

**DOI:** 10.1289/ehp.114-a412

**Published:** 2006-07

**Authors:** Michael Szpir

The etiology of a medical condition might seem an unlikely subject to arouse intense feelings. Yet few medical disorders have stirred up as much passion and divisiveness among scientists and the general public as autism has in recent years. The heat of the controversy has even attracted attention from periodicals such as *The Wall Street Journal*, the *Columbia Journalism Review*, and *Wired* magazine—seemingly improbable forums for a medical debate. Why all the furor?

At the eye of the storm is the startling climb in the numbers of children who have been diagnosed with one of the autism spectrum disorders (ASDs). The most severe ASD is autistic disorder (which often is called simply “autism”); other forms include Asperger syndrome and the much rarer childhood disintegrative disorder. In the United States, the diagnosis of ASDs increased roughly 10-fold over the course of a decade, from 4–5 children per 10,000 in the 1980s to 30–60 children per 10,000 in the 1990s, according to a report in the August 2003 *Journal of Autism and Developmental Disorders*. The 5 May 2006 issue of *Morbidity and Mortality Weekly Report* describes the results of two parent surveys from 2003 and 2004, which suggested that 55–57 children per 10,000 had autism (however, an editorial note points out that, due to the nature of the surveys, parents of children with other ASDs may have reported their children as having autistic disorder).

Some scientists believe that much of the upsurge is the result of increased awareness of ASDs or changes in diagnostic criteria, which would suggest that the true prevalence of the disorders has been stable over time. Others disagree. “It is premature to state that there is no increase in prevalence,” says W. Ian Lipkin, a professor of neurology, anatomy, and neurobiology at Columbia University. “None of the studies to date has been designed to definitively address the issue.”

The prevalence of ASDs plays into the fundamental question of what causes these disorders. If the number of cases is truly on the rise, then it would seem likely that some change in the environment is driving up the total. That’s partly what has divided scientists into opposing camps—they cannot agree on the relative importance of genetic and environmental factors in the disorders’ etiology.

Alas, answering the prevalence question might not end that debate. “Even if the prevalence of autism were stable,” says Lipkin, “you would not be able to rule out the possibility of an environmental trigger.” That’s because very little is known about the mechanisms that cause autism, be they environmental or genetic.

“The study of autism was, until recently, largely dominated by the field of psychology, where characterizing the behaviors and developing reliable instruments for diagnosis have been major areas of research over the past few decades,” says Irva Hertz-Picciotto, an epidemiologist at the University of California, Davis.

Indeed, the core symptoms of ASDs—social disinterest, repetitive and overly focused behavior, and problems in communication, usually appearing before 3 years of age—have been well described. Much less research has focused on the causes of these symptoms.

Several investigations dating back to the 1970s indicate that identical twins have a much higher concordance rate of ASDs than fraternal twins, according to a report in the Spring 1998 issue of *Mental Retardation and Developmental Disabilities Research Reviews*. Those studies provide some of the best evidence that these disorders have a strong genetic component. But the identity of the genes involved, much less how they produce ASDs, has not been established. Moreover, the concordance rate for identical twins is not 100%, which suggests that at least some cases must be associated with environmental or epigenetic factors.

A few cases of ASDs have been clearly linked to environmental insults. These include prenatal exposure to chemical agents such as thalidomide and valproic acid, as well as to infectious agents such as the rubella and influenza viruses. Here again, the concordance rate is not 100%, which suggests that a genetic predisposition is necessary for chemical and microbial factors to act as triggers.

Tantalizing clues like these are prompting scientists to reconsider the research agenda for ASDs. Martha Herbert, a pediatric neurologist at Harvard Medical School, and her colleagues have been applying the methods of genomics to identify environmentally responsive genes that might be important in these disorders.

“When you realize that the widespread changes we’re seeing in autistic brains may occur in parallel with or even downstream from widespread changes in the body—such as in the immune system—and that these changes may be environmentally triggered, you start looking for ways to think more broadly about genetic vulnerability. It can’t be just about ‘brain genes,’” Herbert says.

Some new epidemiological studies also are looking for gene–environment interactions. According to Diana Schendel, an epidemiologist and project officer for autism research at the CDC, which funds one of the projects, these initiatives will be able to examine many possible causal pathways to ASDs, including both genetic and environmental causes that may lead to the development of the disorders in different subgroups of children.

Some of these projects are already under way, whereas others will begin soon. All of the scientists involved, however, believe their research will finally provide some of the answers that everyone has been looking for.

## CHARGE

The Childhood Autism Risks from Genetics and the Environment (CHARGE) project is unique among the large ASD epidemiological studies. It focuses solely on autistic disorder, and it emphasizes a search for environmental factors—including a broad array of chemicals in food, consumer products, and ambient air, as well as infectious and medical exposures—that might be linked to the disorder. The study is funded by the NIH.

CHARGE is a case–control study in which a group of autistic children aged 2 to 5 years is compared to a group of age-matched controls in a population-based study. “Because of the California Department of Developmental Services’ system of Regional Centers [nonprofit corporations that coordinate health care services and support for citizens with developmental disabilities], we have a handle on enumerating a high proportion of the children newly diagnosed with autism in our defined area over a specific time period,” says Hertz-Picciotto, the principal investigator of the CHARGE study. “Similarly, we can enumerate the children in the same area and time period who are not cases. We then sample from both.”

The project was initiated in 2002 with the goal of recruiting 1,000 to 2,000 children. Half of the children will be autistic. The other half will make up two control groups: one group of children with developmental delays (but not an ASD) and a second group of children selected from the general population without regard to developmental characteristics.

The advantage of the case–control design is that scientists can acquire large numbers of children with the disorder. By comparison, in a cohort design researchers would need a very large sample size, given the prevalence of autism, to acquire the same number of cases.

Hertz-Picciotto expects to have enrolled nearly 700 children by August 2006, the end of the first funding period. “I’ve applied for another five-year grant,” she says, “and I hope to be funded to enroll nine hundred in that round, which would bring us to sixteen hundred children.”

The CHARGE team is looking at possible exposures during the prenatal period and early childhood. Some of the data will be gathered through comprehensive interviews with parents, but Hertz-Picciotto admits that this is not the best way to look for exposures. “You ask people questions, and their answers may be colored by the fact that they know they have a child with a condition,” she says. “They may spend a lot of time thinking about what they might have done or what might have gone wrong, and they may have preconceived ideas about what caused [the disorder]. They might not be as objective.” Such problems with postdiagnosis interview information are recognized as a weakness of retrospective studies.

The scientists are getting around this issue by examining each child’s medical records and those of the mother during pregnancy and delivery—nonsubjective data gathered in the course of routine obstetric care. They are also collecting blood, urine, and hair specimens that will be analyzed in the laboratory.

The study has already provided some intriguing leads. “We’re finding that the immune system seems to function at a lower level in autism,” says Hertz-Picciotto. “That’s an important clue. It could mean that whatever causes autism also disrupts the immune system, or it could be that the immune system disrupts neural development so that something goes awry in laying down brain circuitry prenatally or in the early postnatal period.” [For more information on the CHARGE study, see p. 1119, this issue.]

## ABC

The Autism Birth Cohort (ABC) Study, now under way in Norway, is a large prospective design that is expected to gather information on 100,000 babies. The work is being led by scientists at the Mailman School of Public Health at Columbia University, who are collaborating with colleagues at the Norwegian Institute of Public Health, with funding from the U.S. National Institute of Neurological Disorders and Stroke.

“When you want to know why some people are more at risk than others in a population, then that’s best answered using a cohort design,” says Ezra Susser, an epidemiologist at Columbia University and a co-investigator on the ABC project. “When we think about environmental causes of [ASDs], we’re probably interested in phenomena that occur prior to birth or perhaps shortly after birth. So you want to collect prospective data from people as early as possible in pregnancy.” Because ASDs are not common, the study will need large numbers of children to have enough statistical power, according to Susser.

So far the ABC team has recruited 75,000 pregnant Norwegian mothers, but Susser is hoping for more. “We’ve got enough to look for an environmental risk factor, but you need larger numbers for studying gene–environment interactions, which could turn out to be important,” he says. It’s possible the team could acquire greater numbers by collaborating with other studies. One candidate for collaboration is the Avon Longitudinal Study of Parents and Children in the United Kingdom, which is looking at the complex ways in which environmental features may relate to optimal development and health in children. But there’s been no agreement yet, Susser says.

Even so, the ABC scientists are optimistic about their study. “Little is known about the natural history of [ASDs],” says Lipkin, who is the principal investigator of the project. “By starting prenatally, we’re collecting detailed, critical information about environmental exposures in an unbiased fashion.”

The scientists are also collecting plasma, serum, RNA, and DNA. “We have extraordinary biological materials,” says Lipkin. “We can pursue biomarkers as well as exposure to toxicants and infection. We also have maternal DNA, paternal DNA, and the child’s DNA [so-called trio data]; thus we can look for the appearance of novel mutations,” he adds.

The ABC researchers will follow the children through time, with parents answering questionnaires about the health and social interactions of their children as they reach 6, 18, and 36 months of age. “It may be that the developmental trajectory tells us much more than a single time point can ever tell us about the pathogenesis of [ASDs],” says Mady Hornig, a physician-scientist at Columbia University who participates in the project.

Despite their enthusiasm for the project’s potential, the ABC scientists feel they could accomplish much more if they only had the funding. “The pity of it is we have no money to do the biological work,” says Lipkin. “We can collect the samples and do the questionnaires, but we’ve been unable to get funding to look for any of the environmental factors. We’re collecting blood, but we won’t know whether there’s a biomarker until we do a biomarker analysis. We have funds to collect RNA, but in order to do the transcript profiling we need approximately four hundred dollars per sample,” he says.

Lipkin adds that there’s only so much that one can do with questionnaire data. “We do ask about infection and diet, but that’s not the same as having a lab value that can validate what was reported, and then look at a direct correlation with the outcome,” he says.

Lipkin believes that part of the problem is that searching for environmental factors goes against the current research paradigm in ASDs. “The focus is on genetic factors,” he says. “Infectious diseases, toxicology, and immunology receive short shrift. The ABC is clearly the right opportunity to pursue these other leads because we have the ideal samples to survey prenatally and postnatally,” he says.

The scientists are just now receiving the responses to the 36-month questionnaire. “It’ll probably be another two years before we have our first report,” Hornig says. Funds are now in place to study the children at 36 months; however, the team hopes to follow them for a lifetime, according to Hornig.

## CADDRE

In response to the Children’s Health Act of 2000, the CDC established and funds six Centers for Autism and Developmental Disabilities Research and Epidemiology (CADDRE) to investigate potential risk factors for ASDs. The multisite approach offers a study group that is geographically and demographically more representative of the general U.S. population than a smaller regional study could provide, according to Craig Newschaffer, an epidemiologist and principal investigator at the Johns Hopkins Bloomberg School of Public Health CADDRE site.

According to Newschaffer, the CADDRE sites will use a case cohort design in which the exposure patterns of the ASD cases are compared to a random sample of children living in the same geographic area. A third study group, consisting of neurodevelopmentally impaired children who do not have an ASD, will round out the sample populations. The investigators hope to enroll a total of 650 to 900 children, aged 3 to 5 years, in each study group across all the sites, making CADDRE the largest study of its kind in the United States, says Newschaffer. A uniform protocol across the sites will allow the scientists to pool their data.

CADDRE will collect and archive blood, cheek cell, and hair samples from the children in order to investigate a broad range of potential risk factors. “We’re not focused on the environment as much as CHARGE is,” says Newschaffer, “but we are collecting data on questionnaires and reviewing medical records on exposure, in addition to the biosampling for exposures.”

The scientists should have sufficient numbers to look at gene–environment interactions. “We are collecting DNA from the parents and the kids from each of the groups. We’ll have trio data in each of the three groups, a potentially powerful design,” says Newschaffer.

CADDRE scientists will also characterize the behavior of the children, as well as describe any comorbid medical conditions and atypical physical features. The goal is to sort out different etiologic subgroups within the autism spectrum. As Newschaffer explains, “There are a lot of possible reasons why we’ve had a hard time coming up with genetic and nongenetic risk factors. One of them is that autism is likely a heterogeneous condition, with different etiologies producing kids with what appear to be similar phenotypic profiles. If you don’t separate out the different etiologic groups, it’s going to be very hard to find an association with a gene or an exposure. If we limit our analyses to kids that have a certain profile, we’re going to be able to make some informed guesses about what profiles might allow risk factors to emerge,” he says. The CADDRE sites will begin recruiting children into the study in the fall of 2006.

### More Studies, More Acronyms

There are several other smaller epidemiological studies in the works. In California, scientists are tapping into specimen banks that have stored blood samples taken from mothers during pregnancy and from their children at birth. The Early Markers for Autism (EMA) study employs a case–control design, with about 100 children with an ASD (primarily autism), 100 who are developmentally delayed, and 200 from the general population. “We can correlate what’s happening in the mom and the baby, which is really exciting,” says Lisa Croen, a perinatal epidemiologist at the Kaiser Permanente Division of Research in California and the project’s principal investigator.

EMA is a multidisciplinary collaboration with epidemiologists, geneticists, immunologists, neurovirologists, and endocrinologists, according to Croen. “Because autism is so complex, it’s important for all these researchers to communicate with each other. I think EMA is a model for how to do research in autism,” she says. EMA is unique, according to Croen, because the study will be looking for biological markers of ASDs very early in development, during gestation, and at birth. “This allows us to focus on mechanisms that may be leading to autism rather than mechanisms that are consequences of having autism,” she says.

The EMA scientists are investigating genetic and nongenetic factors, with a focus on the immune dysregulation hypothesis of ASDs. “We’re measuring different kinds of immune markers, including immunoglobulin levels and antibodies to specific infectious agents, cytokines, and autoantibodies,” says Croen. “We’re looking for things that distinguish kids who are subsequently diagnosed with autism from those who aren’t. This will help us understand the pathobiology of autism—the mechanisms that are leading to the dysregulation in development.”

The three-year EMA is currently in its last year. “We still have lots of analyses to do,” says Croen, “but we’re beginning to write some papers. We’re finding differences between the children in levels of certain proteins measured in the circulating blood collected from mothers during pregnancy. I think the study has much to contribute to our understanding of the biology of what might be going wrong.”

Croen is also an investigator on the California Autism Twin Study (CATS), which expects to recruit 300 identical and fraternal twin pairs born between 1987 and 1999 in which at least one of the twins has an ASD. Comparing the twin pairs will allow the scientists to estimate the heritability of ASDs—the relative genetic and environmental contributions to the disorder. “Knowing the behavioral and developmental differences between the twins might help us understand the effects of gene expression, the *in utero* environment, and environmental triggers,” Croen says.

Hertz-Picciotto is also excited about a five-year study that she and her colleagues hope to begin soon. Unlike CHARGE, the new effort, called MARBLES (Markers for Autism Risk in Babies—Learning Early Signs), will be a prospective study in which data will be gathered before the children are diagnosed. Pregnant women who already have at least one child with autism will be enrolled right at the beginning of pregnancy. The mothers will keep diaries about their symptoms and health-related events, and the researchers will collect cord blood samples and placentas.

Based on previous research, Hertz-Picciotto expects that about 1 in 10 siblings of the autistic children will also have the disorder, and perhaps 1 in 4 or 5 will be “on spectrum” with a related but less severe condition such as Asperger syndrome, or with some symptoms of the broad behavioral phenotype, such as language delays and atypical social skills. “This work is complementary to the case–control approach, and should provide us with a lot of information that will build on what we find in CHARGE. It should be a phenomenal resource,” she says.

### You Say You Want a Revolution

In April 2004, the U.S. DHHS issued a publication, *Congressional Appropriations Committee Report on the State of Autism Research*, describing recommendations made by a panel of expert scientists convened by the Interagency Autism Coordinating Committee (IACC). The IACC panel suggested an ambitious agenda, which included the goal of identifying environmental risk factors and their associated developmental windows within a four- to six-year period, as well as identifying genetic and nongenetic causes of ASDs and their interactions within seven to ten years.

Hertz-Picciotto, a member of the IACC panel, thinks these goals should be taken with a grain of salt. “I’m optimistic that we will have identified some environmental risk factors, and may have excluded a few others, between 2008 and 2010—but by no means will we have the final word. The genetics and the gene–environment interactions may be even tougher. Unfortunately, I don’t see enough groups working on the environmental contribution to autism, so it may be slower than projected,” she says.

Mark Blaxill, vice president of SafeMinds, a parent-led advocacy group, also believes that environmental risk factors don’t receive enough consideration. “The CDC has not addressed the crisis in autism responsibly,” he says. “They should be raising the alarm, and they have failed to do so. They should be asking why so many children are sick. Instead, they’ve tried to suggest a degree of doubt about the increases, and that diverts attention and funding from environmental causes.”

Schendel responds, “It is clear that more children than ever before are being classified as having an ASD. It is important that we treat common developmental disorders, and especially the ASDs, as conditions of urgent public health concern. The CDC’s efforts in addressing this public health concern include funding for ASD monitoring programs to understand ASD trends, funding for research into the genetic and environmental causes of ASDs, and education and outreach programs to promote early identification and timely intervention for all children with developmental problems.”

Despite the promise of the new epidemiological studies, some researchers are still dismayed, as one scientist put it, that “geneticists are running the show, and ignoring the environmental aspects.” What would it take for things to change? Blaxill invokes the ideas of philosopher Thomas Kuhn, who suggested that scientific revolutions occur when an old paradigm is replaced by a new one. “I believe we’re in the middle of a paradigm shift,” Blaxill says. “The dramatic explosion of autism rates does not fit the genetic model. It’s an anomaly that will kill the old paradigm.”

## Figures and Tables

**Figure:**
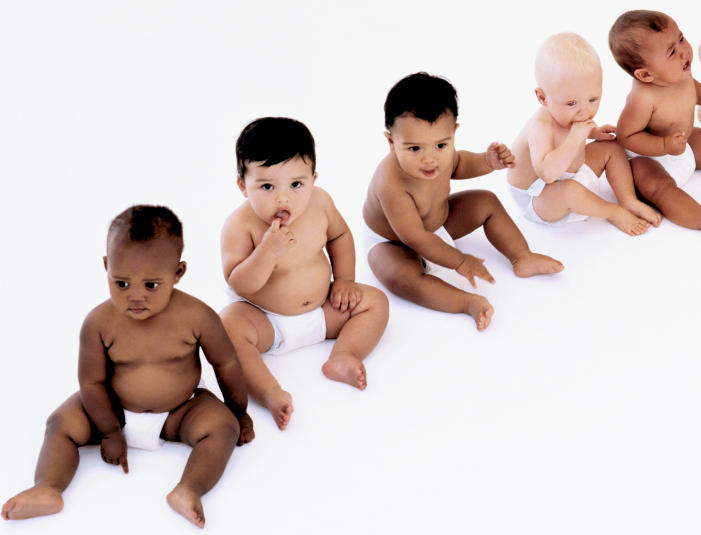


**Figure:**
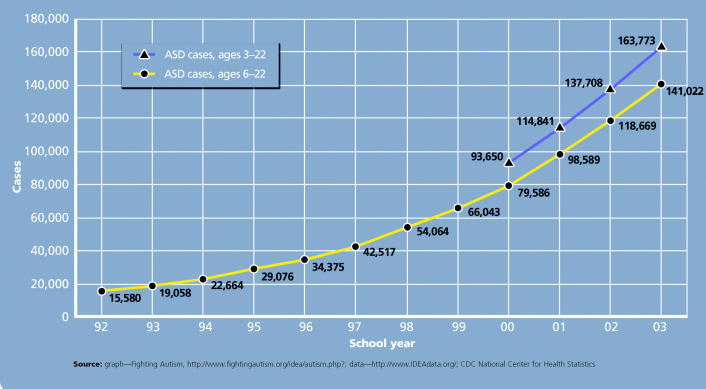
Reported Cases of ASDs in the United States and Outlying Areas

**Table:** Studies of Environmental Factors in Autism Spectrum Disorders (ASDs)

**Name of Study** (Location)	**Goal**	**Study Size**	**Time Frame**	**Ages Studied**	**Funding Source(s)**
**Autism Birth Cohort** (Norway)	Investigate prenatal and postnatal environmental exposures that may lead to ASDs	100,000	2004–2008	Gestation– 3 years	Columbia University, Norwegian Insititute of Public Health, NINDS
**California Autism Twin Study** (United States)	Study the behavior and learning styles of children with autism and their twins	300 twin pairs	2004–2009	Not specified	NIMH
**Centers for Autism and Developmental Disabilities Research and Epidemiology** (United States)	Compare environmental exposure patterns of children with ASDs, neurodevelopmentally impaired children without ASDs, and the general population	2,700	2000–2011 (to date)	3–5 years	NIH
**Childhood Autism Risks from Genetics and the Environment** (United States)	Investigate prenatal and early childhood environmental exposures that may contribute to ASDs	2,000	2002–2006 (possible 5-year extension)	2–5 years	NIH
**Early Markers for Autism** (United States)	Analyze maternal and infant blood samples for early biomarkers of ASDs	400	2004–2006	Gestation– 3 years	NIMH, National Alliance for Autism Research
**Markers for Autism Risk in Babies—Learning Early Signs** (United States)	Study prenatal factors that may affect development of ASDs in children with at least one sibling with an ASD	unknown	2006–2011 (planned)	Gestation– unknown	unknown

**Key to U.S. Funding Agencies:** NIMH—National Institute of Mental Health; NINDS—National Institute of Neurological Disorders and Stroke; NIH—National Institutes of Health

**Table:** Other Major Environmental Health–Related Studies

**Name of Study** (Location)	**Goal**	**Study Size**	**Time Frame**	**Ages Studied**	**Funding Source(s)**
**Agricultural Health Study** (United States)	Evaluate the role of agricultural exposures in the development of cancer and other diseases in the farming community	90,000	1993–2008	Children, adults	NCI, NIEHS, EPA
**Australian Multi-Centre Study of Environment and Immune Function**	Examine how environmental factors influence immune diseases and how immune disorders vary by latitude across Australia	1,000	2003–2008	Teenagers, adults	National Multiple Sclerosis Society (U.S.)
**Avon Longitudinal Study of Parents and Children** (United Kingdom)	Determine the current problems in child health and development and how they may be prevented	14,000	1991–2010	Infant–early adulthood	UK Medical Research Council, Wellcome Trust, others
**Bangladesh Vitamin E and Selenium Trial**	Investigate whether vitamin E and/or selenium has a beneficial effect in reducing skin cancers and other types of cancer	4,500	2005–2010	25–65 years	NIH
**Diesel Particle Exposure and Lung Cancer** (United States)	Assess the association between exposure to diesel exhaust and lung cancer mortality	55,750	2001–2007	Adults	NCI
**French Longitudinal Study of Children**	Describe child growth at different ages, assess levels of exposure to the main environmental pollutants, and analyze the links between exposure and public health	20,000	2005–undetermined	Birth– adulthood	French government, others
**GABRIEL—A Multidisciplinary Study to Identify the Genetic and Environmental Causes of Asthma in the European Community**	Examine the roles of genetic and environmental factors influencing the development of asthma	40,000	2006–2009	Children, adults	European Commission
**Gene–Environment Interactions in Facial Clefts** (Denmark, Norway)	Use advances in molecular technologies to provide a new level of understanding for a complex birth defect trait	200,000	1998–2007	Infants	NIDCR
**Genetic and Environmental Influences on Childhood Growth** (Nepal)	Elucidate the roles of genetic and environmental factors influencing childhood growth and development	900	2002–2007	3–18 years	NICHD
**Health Effects of Arsenic Longitudinal Study** (Bangladesh)	Prospectively examine the health effects of arsenic among a population chronically exposed to the chemical through contaminated drinking water	15,000	2000–2011	18–75 years	NIH
**Longitudinal Study of Australian Children**	Assess emerging health and developmental concerns and their determinants in children	10,000	2003–2009	Infant–12 years	Australian government
**National Children’s Study** (United States)	Examine the effects of environmental influences on the health and development of children	100,000	2000–2006 (funding discontinued after 2007)	Gestation– 21 years	NICHD, NIEHS, EPA, CDC
**NewGeneris** (European Union)	Investigate exposure to chemicals in food and the environment and their connection with childhood cancer and immune disorders	600,000	2006–2001	Birth–7 years	European Community
**Swiss Study on Childhood Allergy and Respiratory Symptoms with Respect to Air Pollution, Climate, and Pollen**	Investigate the association between long-term exposure to air pollution and respiratory health and allergies in children	17,846	1997–2008	6–14 years	Swiss government
**Singapore Cohort Study of Diet and Cancer/Singapore Chinese Health Study**	Elucidate the role of diet and its interaction with genetic factors in the causation of human cancer	63,257	1999–2010	45–74 years	NCI, NIEHS
**Sister Study** (United States)	Learn how the environment and genetics affect the chances of getting breast cancer	50,000	2003–2013	35–74 years	NIEHS
**Southern Community Cohort Study** (United States)	Gain new information about the causes of cancer, heart disease, and other common illnesses	100,000	2002–2007	40–79 years	NCI
**The Environmental Determinants of Diabetes in the Young** (United States, Finland, Germany, Sweden)	Identify infectious agents, dietary factors, or other environmental agents, including psychosocial factors, that trigger type 1 diabetes mellitus	7,092	2004–2023	Infant–15 years	NIDDK, NIAID, NICHD, NIEHS, CDC, JDRF

**Key to U.S. Funding Agencies:** CDC—Centers for Disease Control and Prevention; EPA—Environmental Protection Agency; JDRF—Juvenile Diabetes Research Foundation; NCI—National Cancer Institute; NIAID—National Institute of Allergy and Infectious Diseases; NICHD—National Institute of Child Health and Human Development; NIDCR—National Institute of Dental and Craniofacial Research; NIDDK—National Institute of Diabetes and Digestive and Kidney Diseases; NIEHS—National Institute of Environmental Health Sciences; NIH—National Institutes of Health

